# Effects of S‐pindolol in mouse pancreatic and lung cancer cachexia models

**DOI:** 10.1002/jcsm.13249

**Published:** 2023-05-02

**Authors:** Jochen Springer, Queralt Jové, Edson Alves de Lima Junior, Natalia Álvarez Ladrón, Francisco Javier López‐Soriano, Silvia Busquets, Josep M. Argiles, Daniel L. Marks

**Affiliations:** ^1^ BIH Center for Regenerative Therapies Charité University Medical Center Berlin Berlin Germany; ^2^ German Centre for Cardiovascular Research (DZHK) partner site Berlin Charité Universitätsmedizin Berlin Berlin Germany; ^3^ Departament de Bioquímica i Biomedicina Molecular, Cancer Research Group, Facultat de Biologia, Institut de Biomedicina de la Universitat de Barcelona (IBUB) Universitat de Barcelona Barcelona Spain; ^4^ Oregon Health & Science University Portland Oregon USA

**Keywords:** body weight, cancer cachexia, grip strength, lung cancer, pancreatic cancer, S‐pindolol

## Abstract

**Background:**

It is known that S‐pindolol attenuates muscle loss in animal models of cancer cachexia and sarcopenia. In cancer cachexia, it also significantly reduced mortality and improved cardiac function, which is strongly compromised in cachectic animals.

**Methods:**

Here, we tested 3 mg/kg/day of S‐pindolol in two murine cancer cachexia models: pancreatic cancer cachexia (KPC) and Lewis lung carcinoma (LLC).

**Results:**

Treatment of mice with 3 mg/kg/day of S‐pindolol in KPC or LLC cancer cachexia models significantly attenuated the loss of body weight, including lean mass and muscle weights, leading to improved grip strength compared with placebo‐treated mice. In the KPC model, treated mice lost less than half of the total weight lost by placebo (−0.9 ± 1.0 vs. −2.2 ± 1.4 g for S‐pindolol and placebo, respectively, *P* < 0.05) and around a third of the lean mass lost by tumour‐bearing controls (−0.4 ± 1.0 vs. −1.5 ± 1.5 g for S‐pindolol and placebo, respectively, *P* < 0.05), whereas loss of fat mass was similar. In the LLC model, the gastrocnemius weight was higher in sham (108 ± 16 mg) and S‐pindolol tumour‐bearing (94 ± 15 mg) mice than that in placebo (83 ± 12 mg), whereas the soleus weight was only significantly higher in the S‐pindolol‐treated group (7.9 ± 1.7 mg) than that in placebo (6.5 ± 0.9). Grip strength was significantly improved by S‐pindolol treatment (110.8 ± 16.2 vs. 93.9 ± 17.1 g for S‐pindolol and placebo, respectively). A higher grip strength was observed in all groups; whereas S‐pindolol‐treated mice improved by 32.7 ± 18.5 g, tumour‐bearing mice only show minimal improvements (7.3 ± 19.4 g, *P* < 0.01).

**Conclusions:**

S‐pindolol is an important candidate for clinical development in the treatment of cancer cachexia that strongly attenuates loss of body weight and lean body mass. This was also seen in the weight of individual muscles and resulted in higher grip strength.

## Introduction

S‐pindolol is the S‐enantiomer of the racemic, non‐specific beta‐blocker pindolol. It blocks the beta‐1 adenoreceptor, is a partial agonist on the anabolic beta‐2 adenoreceptor and has central effects on the 5‐HT_1α_ receptor, which likely contributes to higher food intake.^1^ In previous experiments, S‐pindolol showed great efficacy in protecting muscle mass in the Yoshida AH‐130 hepatoma cancer cachexia model^1^ and it increased muscle mass in aging rats.^2^ It attenuated the overall loss of body weight and improved survival significantly in the Yoshida model. The 3 mg/kg/day dose was found to be the most effective in these experiments^1^ and was therefore be used in the experiments described here. Moreover, S‐pindolol was used in a phase IIa clinical study with patients suffering from cachexia due to stage III and IV non‐small‐cell lung cancer or colorectal cancer. Patients treated with 10 mg of S‐pindolol twice daily significantly reversed weight loss, improved fat free mass and maintained fat mass. This was associated with a significant improvement in hand grip strength.^3^, ^4^

The aim of the study was the confirmation that 3 mg/kg/day of S‐pindolol also shows efficacy in a second species (mouse) and additional cancer cachexia models (pancreatic cancer cachexia [KPC] and Lewis lung carcinoma [LLC]).

## Methods

Male C57BL/6J mice were 7–12 weeks of age and 23.9 ± 0.6 g in weight for KPC and 17.5 ± 0.4 g in weight for LLC at the start of experimental procedures. Thirty‐one mice were randomized to sham (*n* = 8) or orthotropic tumour inoculation (*n* = 23) of 3 million KPC cells in a volume of 40 μL into the tail of the pancreas, as described before.^5^ Tumour‐bearing mice were further randomized to treatment with placebo (*n* = 11) or 3 mg/kg/day of S‐pindolol (*n* = 12) once daily for 13 days. Body composition, that is, lean and fat mass, was assessed by nuclear magnetic resonance (NMR) spectroscopy (Echo‐MRI 700 TM, Echo Medical Systems, Houston, TX, USA) 1 day before tumour cell inoculation and on the day of euthanasia.

For the LLC model, 30 C57BL/6 mice (mean body weight of 17.5 ± 0.4 g) were randomized to treatment: no tumour injection, sham (*n* = 5), placebo (sterilized water, *n* = 15) or 3 mg/kg/day of S‐pindolol (*n* = 10) for 13 days. Mice received an intramuscular (hind leg) inoculum of 5 × 10^5^ LLC cells obtained from exponential tumours, as described before.^6^, ^7^ Grip strength test was performed by a pull bar connected to an isometric force transducer (dynamometer, Panlab‐Harvard Apparatus, Spain). The animals were allowed to grasp the bar and were then pulled backwards in the horizontal plane.^8^

In both models, S‐pindolol was given once daily by gavage. All procedures were approved by the local animal ethics committee in Portland (OHSU IACUC Approval TR03_IP00000038) and Barcelona (Generalitat de Catalunya, Nos 10055 and 229.18, University of Barcelona).

Results, represented as mean ± SD, were analysed using GraphPad Prism 8.0 (GraphPad Software, Inc., La Jolla, CA, USA). All data were tested for normal distribution using the Kolmogorov–Smirnov test. Group comparisons were performed by one‐way analysis of variance (ANOVA) with adjustment for multiple testing of sham‐treated controls and S‐pindolol‐treated tumour‐bearing animals against tumour‐bearing vehicle controls. A *P*‐value of <0.05 was considered significant.

## Results

### Pancreatic cancer cachexia model

**Figure 1:**
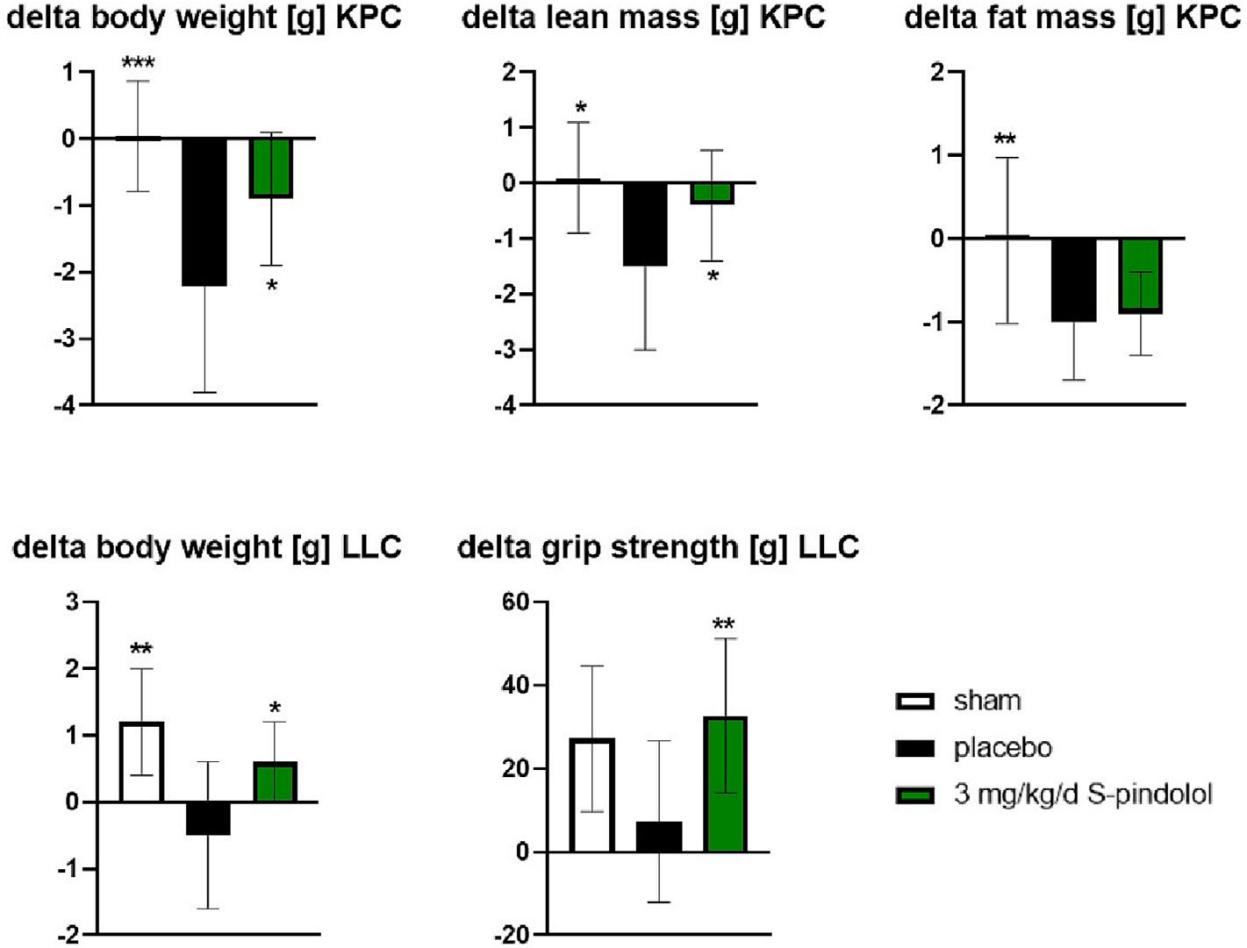
Shown are the change in body weight, lean and fat mass from baseline to the end of the study in the pancreatic cancer cachexia (KPC) model and the change in body weight and grip strength in the Lewis lung carcinoma (LLC) model. *P < 0.05, **P < 0.01 and ***P < 0.001 versus placebo.

Sham‐treated controls maintained their body weight and proportion of lean and fat mass over the study as would be expected. Tumour size/weight was not different between placebo (1.1 ± 0.6 g) and S‐pindolol‐treated animals (1.3 ± 0.8 g). In contrast to sham, tumour‐bearing vehicle controls lost around 10% of their initial body weight over the study and showed falls in mean lean and fat mass of 1.5 and 1 g, respectively. Mice treated with 3 mg/kg/day of S‐pindolol lost less than half of the total weight lost by tumour‐bearing controls (−0.9 ± 1.0 vs. −2.2 ± 1.4 g for S‐pindolol and placebo, respectively, *P* < 0.05) and around a third of the lean mass lost by tumour‐bearing controls (−0.4 ± 1.0 vs. −1.5 ± 1.5 g, *P* < 0.05). Fat loss in S‐pindolol‐treated mice was comparable with that in tumour‐bearing controls (*Figure* *1* and *Table* *1*).

**Table 1:** Mean values with statistical analysis for body weight, fat and lean mass with change (delta from baseline to the end of the study), and food intake and tissue weights in the pancreatic cancer cachexia model

	Sham Vehicle	Tumour‐bearing	
		Vehicle	3 mg/kg/day of S‐pindolol
*n*	8	11	12
Tumour (g)	0	1.1 ± 0.6	1.3 ± 0.8
Body weight, Day 0 (g)	24.3 ± 1.3	24.4 ± 0.8	24.5 ± 1.0
Final body weight (g)	24.4 ± 1.7*	22.2 ± 1.6	23.6 ± 1.5
Delta body weight (g)	0.04 ± 0.83***	−2.2 ± 1.4	−0.9 ± 1.0*
Lean, Day 0 (g)	21.4 ± 1.2	21.3 ± 1.1	21.5 ± 1.0
Final lean (g)	21.4 ± 1.4*	19.8 ± 1.3	21.0 ± 1.4*
Delta lean (g)	−0.1 ± 1.0*	−1.5 ± 1.5	−0.4 ± 1.0*
Fat, Day 0 (g)	1.8 ± 0.3	1.9 ± 0.4	1.8 ± 0.2
Final fat (g)	1.8 ± 0.4***	0.9 ± 0.5	1.0 ± 0.5
Delta fat (g)	−0.02 ± 0.30**	−1.0 ± 0.7	−0.9 ± 0.5
Cumulative food intake, Days 7–13 (g)	17.87 ± 1.25*	14.57 ± 3.45	17.37 ± 1.65*
Heart (mg)	109.7 ± 18.9*	96.1 ± 6.3	96.8 ± 7.9
Spleen (mg)	79.9 ± 9.1*	121.7 ± 22.8	121.4 ± 21.2
Gastrocnemius (mg)	140.9 ± 10.4**	122.1 ± 12.6	140.3 ± 13.6**
Soleus (mg)	7.5 ± 0.5*	6.6 ± 1.0	7.4 ± 0.7*

* *P* < 0.05 versus tumour‐bearing vehicle controls.

** *P* < 0.01 versus tumour‐bearing vehicle controls.

*** *P* < 0.001 versus tumour‐bearing vehicle controls.

The protection of body weight in S‐pindolol‐treated animals relative to tumour‐bearing controls was accompanied by improved food intake, which was higher than in tumour‐bearing controls (*P* < 0.05) and similar to that of sham‐treated controls (*Table* *1*).

The mean heart weight of tumour‐bearing controls was around 10% lower than that of sham controls (*P* < 0.05), whereas their spleen weight was 53% higher (*P* < 0.05). S‐pindolol‐treated animals and sham controls showed near identical differences to tumour‐bearing placebo mice for these organs. In contrast, whereas the weights of gastrocnemius and soleus muscles in tumour‐bearing controls were notably lower than in sham controls (*P* < 0.05 and *P* < 0.01, respectively), these muscles in S‐pindolol‐treated animals were of near identical weight to those of sham‐treated controls and so higher than in tumour‐bearing controls (140.3 ± 13.6 vs. 122.1 ± 12.1 mg, *P* < 0.01, for gastrocnemius; 7.4 ± 0.7 vs. 6.6 ± 1.0 mg, *P* < 0.05, for soleus muscle). These results show good conservation of skeletal muscle mass by S‐pindolol (*Table* *1*).

### Lewis lung carcinoma model

Sham‐treated controls increased their body weight by 1.2 ± 0.8 g. Tumour size/weight was not different between placebo (4.4 ± 0.9 g) and S‐pindolol‐treated animals (4.6 ± 0.8 g). Tumour‐bearing vehicle controls lost around 3% of their initial body weight (−0.5 ± 1.1 g), whereas mice treated with 3 mg/kg/day of S‐pindolol gained 3% (0.6 ± 0.6 g, *P* < 0.05) and showed increased grip strength (*Figure* *1* and *Table* *2*). Cumulative food intake was non‐significantly higher in tumour‐bearing mice regardless of treatment allocation. Heart weight was similar in sham and tumour‐bearing controls and increased in S‐pindolol‐treated animals. The mean spleen weight was increased in tumour‐bearing animals, and the gastrocnemius weight was significantly higher in sham (108 ± 16 mg, *P* < 0.01) and S‐pindolol tumour‐bearing (94 ± 15 mm, *P* < 0.05) mice than that in placebo (83 ± 12 mg), whereas the soleus weight was only significantly higher in the S‐pindolol‐treated group (7.9 ± 1.7 mg, *P* < 0.05) than that in placebo (6.5 ± 0.9 mg; *Table* *2*).

**Table 2:** Mean values with statistical analysis for body weight and grip strength with change (delta from baseline to the end of the study), and food intake and tissue weights in the Lewis lung carcinoma model

	Sham Vehicle	Tumour‐bearing	
		Vehicle	3 mg/kg/day of S‐pindolol
*n*	5	15	10
Tumour (g)	0	4.4 ± 0.9	4.6 ± 0.6
Body weight, Day 0 (g)	18.3 ± 2.3	17.3 ± 2.3	17.4 ± 1.9
Final body weight (g)	19.5 ± 1.7*	16.8 ± 1.9	18.0 ± 1.9
Delta body weight (g)	1.2 ± 0.8**	−0.5 ± 1.1	0.6 ± 0.6*
Grip strength, Day 0 (g)	92.1 ± 6.2	86.6 ± 16.6	78.1 ± 9.4
Grip strength, Day 14 (g)	119.3 ± 16.6*	93.9 ± 17.1	110.8 ± 16.1*
Delta grip strength (g)	27.2 ± 17.5	7.3 ± 19.4	32.7 ± 18.5**
Cumulative food intake, Days 0–14 (g)	44.4 ± 5.1	54.7 ± 9.5	52.2 ± 10.9
Heart (mg)	111 ± 11	110 ± 17	125 ± 13*
Spleen (mg)	92 ± 64***	217 ± 39	224 ± 25
Gastrocnemius (mg)	108 ± 16**	83 ± 12	94 ± 15*
Soleus (mg)	7.4 ± 0.9	6.5 ± 0.9	7.9 ± 1.7*

* *P* < 0.05 versus tumour‐bearing vehicle controls.

** *P* < 0.01 versus tumour‐bearing vehicle controls.

*** *P* < 0.001 versus tumour‐bearing vehicle controls.

## Discussion

Treatment of mice with 3 mg/kg/day of S‐pindolol in KPC or LLC cancer cachexia models significantly attenuated the loss of body weight and, in particular, protected lean mass and muscle weights, leading to an improved grip strength compared with placebo‐treated mice. Better muscle function, that is, hand grip strength, is an indicator not only for improved quality of life but also for survival.^9^, ^10^

This study shows that S‐pindolol's positive effects are not limited to a rat model of liver cancer‐associated cachexia.^1^ The effects in the pancreatic cancer (KPC) model are particularly encouraging, as there are extremely limited options for patients with pancreatic cancer, with survival having increased only marginally over the last decades. The 5‐year relative survival rates in the United States have improved only modestly from 2.0% (95% confidence interval [CI], 1.7–2.4%) for patients with pancreatic cancer between 1975 and 1979 to 9.2% (95% CI, 8.7–9.8%) between 2007 and 2011.^11^ A second study puts the changes in age‐standardized mortality rates for all cancer and individual cancer sites at 10.7 in 1971 and at 11.0 in 2019, leading to a ratio of 1.03 (1.01–1.05).^12^ We propose that S‐pindolol may be an important future co‐treatment to stabilize the metabolic homeostasis allowing for improved tolerance of cancer treatments, while attenuating loss of quality of life in this strongly suffering patient population.

In conclusion, S‐pindolol has shown encouraging pre‐clinical and early clinical data, not only in the Yoshida, KPC and LLC in rodent models but also in an early human phase IIa study that included patients with colorectal and non‐small‐cell lung cancer,^3^ making further clinical development imperative.

## Conflict of interest statement

JS is a shareholder of Actimed Therapeutics and a paid consultant for Actimed Therapeutics, Pephexia Therapeutics and Drexel Pharma Technologies and received grant support from Boehringer Ingelheim. JMA declares having received honoraria and/or lecture fees from Amgen, Novartis, Smartfish, Rottapharm/Madaus, Numico, Danone, Grunenthal, Fresenius, Merck KGaA, Merk Sharp & Dohme, Serono, Procter and Gamble, Santhera Pharmaceuticals, Bristol Myers Squibb and Nutricia. DLM is a shareholder and Chief Medical Officer for Endevica Bio, Inc., and a consultant for Pfizer, Inc. All other authors have no conflict of interests.
